# Influence of using simulated or real patients on undergraduate medical students acquiring competencies in medical conversations in surgery: A prospective, controlled study

**DOI:** 10.3389/fsurg.2022.986826

**Published:** 2022-09-12

**Authors:** Vanessa Britz, Yannic Koch, Teresa Schreckenbach, Maria Christina Stefanescu, Uwe Zinßer, Jasmina Sterz, Miriam Ruesseler

**Affiliations:** ^1^ Medical Faculty, Frankfurt Interdisciplinary Simulation Center FIneST, Goethe University Frankfurt/Main, Frankfurt, Germany; ^2^ Department of General, Visceral, Transplantation and Thoracic Surgery, Goethe University Frankfurt/Main, University Hospital Frankfurt, Frankfurt, Germany; ^3^ Department of Pediatric Surgery, University Medical Center of the Johannes Gutenberg University Mainz, Mainz, Germany; ^4^ Department of Trauma-, Hand- and Reconstructive Surgery, University Hospital Frankfurt, Goethe University, Frankfurt, Germany

**Keywords:** simulated patients, medical education, medical conversations, undergraduate students, surgical education

## Abstract

**Background:**

Communication with patients and their relatives as well as with colleagues and students is an essential part of every physician's daily work. An established method for teaching communication skills is using simulated patients (SPs). However, teaching with SPs is often subjectively perceived by medical students as less instructive than teaching with real patients (RPs). Studies that analyze the influence of SPs compared to RPs for acquiring competencies are lacking. The aim of the present study was therefore to investigate the impact of SPs on long-term learning success for communication skills compared to RPs.

**Material and Methods:**

Study participants were undergraduate third-year medical students who attended a communication unit and were randomized into three groups. The first group trained the role-play part with a SP (SP-group). The second group trained with a SP but thought that the patient was a RP because the students and the tutors were told that they were a RP by the principal investigator (incognito patient group [IP-group]). The third group and their tutors trained with a RP and were told that the patient was a RP (real patient group [RP-group]). Five to 12 weeks after completing the training, the study participants completed a curricular summative objective standardized clinical examination.

**Results:**

There were 146 students who participated in the study. There were no significant differences between the three study groups at the informed consent stations and for those conducting anamnesis interviews.

**Conclusion:**

Communication skills training with SPs appears to be equivalent to training with RPs in terms of competency development in communication-based assessments in surgery. Therefore, SPs should be used in these curricula, especially at an early stage, to enable the students to practice adequate communication skills.

## Introduction

Communication with patients and their relatives as well as with colleagues and students is an essential part of every physician's daily work. As early as 1958, Lauda showed that up to 70% of all diagnoses can be made with an adequate anamnesis (1). A well-taken anamnesis can currently at best save unnecessary diagnostics that have no therapeutic effect.

Communication skills are important in every medical discipline that deals with direct patient contact but especially in Urology. Besides the common requirements such medical history taking and leading informed consent discussions in a language that the patient can understand, sensitive topics such as potency, sexuality, or continence also have to be discussed. Although Ernstmann et al. showed that good doctor–patient communication in Urology can improve treatment success (2), Urology residents are confronted with challenging communication situations, such as conversation with patients who have a demanding attitude or emotionally stressful situations where they can sometimes be overwhelmed (3). They, therefore, suggest that learning objectives for communication skills should be integrated into undergraduate and postgraduate medical training (3).

An established method for communication skills training is to use simulated patients (SPs). To support clinical skill learning, the concept of SPs was first presented by Barrows and Abrahamson in 1964 (4). SPs have now been established in almost all medical universities in German-speaking countries (Germany, Austria, and Switzerland) (5). These SPs are defined as “lay people who are trained to portray a patient with a specific condition in a realistic way, sometimes in a standardized way (where they give a consistent presentation, which does not vary from student to student)” (6).

Numerous studies show that SPs are appreciated and accepted by students (7–9). Good acceptance of SPs and the positive influence of SP use on knowledge acquisition and behavior change in learners has been proven (9, 10). For example, Zabel et al. showed that undergraduate medical students performed a knee or shoulder examination significantly better if they trained this examination on a SP who provided feedback than if they trained together under the supervision of a surgeon (11). Additionally, Herbstreit et al. showed that medical students performed slightly but significantly better when handling medical emergencies after they were trained using SPs compared with a traditional seminar cohort (12).

However, teaching with SPs is often subjectively perceived by medical students as less instructive than teaching with real patients (RPs). Generally, students considered RP interactions to be more instructive than interactions with SPs (7, 13). In a qualitative study that included 38 medical students, Bokken et al. showed that these students perceived RP encounters as more instructive and more authentic than conversations with SPs. The participants in this study also described that they saw the conversations with SPs as good preparation for conversations with RPs and that particularly difficult situations such as dealing with angry patients can be practiced well with SPs (7). However, studies that have analyzed not only the evaluations of the students, but also the influence of SPs compared to RPs on the acquisition of competencies are lacking.

The aim of the present study was to investigate the impact of using SPs on long-term learning success in communication skills compared to RPs.

## Material and methods

### Participants and background

This study had a prospective design and was performed in accordance with the ethical principles of the World Medical Association Declaration of Helsinki (Ethical Principles for Medical Research Involving Human Subjects). It was also reviewed by the ethics committee of the University Hospital of Frankfurt (Johann Wolfgang Goethe University), which indicated that no further approval was required.

Study participants were third-year undergraduate medical students. These students participated in the study after they received a detailed oral and written explanation of the study and provided written informed consent. Participation in the study was voluntary and could be terminated at any time without explanation. Epidemiologic data from each participant were gathered before the start of the study.

The study was performed during the mandatory 1-week long surgical skills lab training (14). This training contained 12 teaching units for practical basic surgery skills such as communication training and a teaching unit on abdominal ultrasound. The prerequisite for participation in the skills lab training was attendance at the main lecture on surgery and passing the associated written exam. The skills lab training was followed by a 2-week surgical internship.

### Communication unit

The study took place during the 210 min of the communication training unit and aimed for the students to correctly take a patient's medical history and to meaningfully structure informed consent discussions for surgical interventions. The unit was conducted by student peer tutors. The quality of the units was monitored and maintained using tutor manuals, standardized presentations, and mandatory tutor training on a regular basis.

The teaching unit began with the students working out the essential components involved in taking a medical history under the guidance of the tutors before they practiced taking a patient's history. After the exercise, the students received 360° feedback on the content and on how they treated the patient. Special focus was placed upon communication competencies such as empathetic behavior and strategies to deal with sensitive topics. The second part focused on informed consent discussions and was conducted in the same manner. At the end of the unit, each student had at least taken one history or led an informed consent discussion.

### Intervention

Regardless of study participation, students were assigned to groups by the Office of the Dean before the study. Bias was reduced because the principal investigator was not involved in the group assignments. Individual randomization was not possible because this study was integrated into the curriculum.

The participants attended the communication unit according to their group allocation. Both the theoretical part and the time limit were identical for all groups. The first group trained the role-play part using SPs (SP-group), and the participants were informed that SPs were used. The second group trained with a SP, but they thought that the patient was a RP because the students and the tutors were told that they were RPs by the principal investigator (incognito patient group [IP-group]). The third group together with their tutors trained with a RP and were correctly informed about it (real patient group [RP-group]).

### Measurement

Five to 12 weeks after completing the training, the study participants completed a curricular summative objective standardized clinical examination (OSCE). During the study period, this OSCE consisted of eight stations and three to four of these stations were communication-based (taking a patient’s medical history or obtaining informed consent before surgery). Each station included 2 min to read the task and 5 min to solve the task.

The individual stations were evaluated using a two-part evaluation sheet. On the one hand, the station consisted of a standardized content-related checklist (part A). Individual content-related items (e.g., question about allergies and the nature of the pain) were evaluated using a three-part scale (0 = not performed/asked, 1 = partially/incorrectly performed/asked, 2 = correctly performed/completely asked). The total number of items differed depending on the task. The second part of the evaluation sheet assessed the overriding aspects of the interaction with the patient, such as communicating in language that was understandable to the patient or responding to the patient's questions. Five items were evaluated on a five-point Likert scale using fixed anchor criteria for 1, 3, and 5 points. For the overall assessment of the individual stations, the first part was weighted as two-thirds and the second part as one-third of the score. The evaluation sheets used in the present study were primarily piloted in previous undergraduate training. The content validity was ensured through the creation as part of an expert workshop with didactic and surgical experts as well as through the repeated application and adaption in the context of previous OSCE exams.

The examiners were surgeons who participated in the OSCE as part of their regular teaching duties. Examiner training was mandatory to become an examiner. This included training on how to use the checklists as well as taking a neutral role in the background. Examiners were blinded to the students' group allocation.

### Statistical methods

Data were processed using Microsoft Excel (Microsoft Inc., Redmond, WA, USA). Statistical analysis was performed using IBM SPSS version 24 (IBM Corp., Armonk, NY, USA).

Tests between the groups were calculated using a parametric variant analysis of variance (ANOVA). If variant homogeneity was present, *p*-values for comparing groups were analyzed using Tukey's test. For variant heterogeneity, a corrected ANOVA (Welch's test) was used, and for *p*-value analyses between groups, the Games–Howell test was applied. Significance was defined as *p* < 0.05. An *a priori* sample size calculation was performed using G * Power (University Düsseldorf, Düsseldorf, Germany). Assuming a mean effect size of f = 0.4, the required group size was 34.

## Results

One hundred forty-six students agreed to participate in the study, and 38.36% of them were men, while the mean age was 22.9 ± 2.8 years. The study group is thus representative of the sixth semester at the Goethe University in Frankfurt, Germany where the study was conducted. Table 1 shows the composition of the three study groups. Among the 146 participants, 131 (89.73%) completed the summative OSCE. The remaining students either did not meet the qualifications for the final examination or did not return.

**Table 1:** Epidemiological data for the study groups.

Group	1	2	3	Total
*N (Training)*	49	44	53	146
*N (OSCE)*	39	42	50	131
Male (*n*) (%)	21 (42.86)	12 (27.27)	23 (43.4)	56 (38.36)
Age (years)^a^	22.8 ± 2.8	22.5 ± 2.1	23.2 ± 3.3	22.9 ± 2.8
Number of Semesters^a^	6.4 ± 0.8	6.2 ± 0.8	6.4 ± 1.0	6.3 ± 0.9

^a^ Mean ± standard deviation.

**Figure 1:**
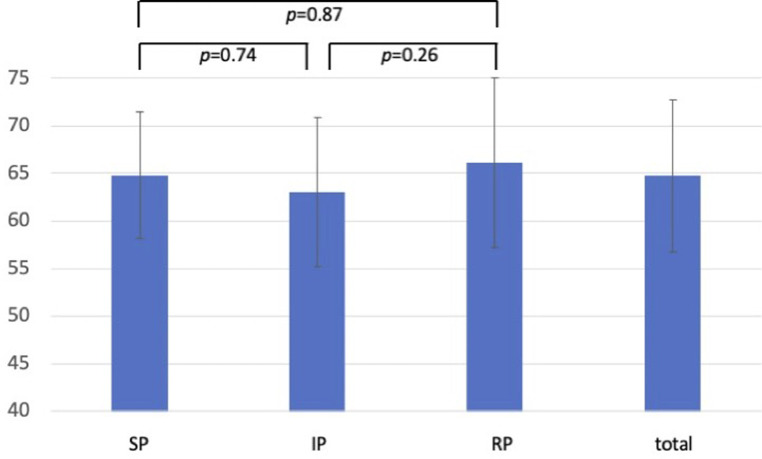
OSCE results for content-related items (first part of the evaluation sheet). Results of the rating for the content-related items. Data are presented as the mean ± standard deviation of the achieved results as a percentage. (SP-group, simulated patient group; IP-group, incognito patient group; RP-group, real patient group).

**Figure 2:**
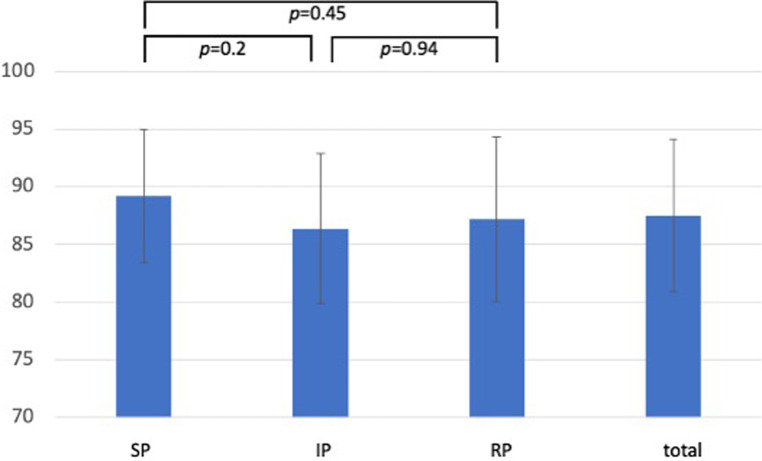
OSCE results for the second part (aspects of interaction) of the evaluation sheet. Results of the rating for aspects of interaction. Data are presented as the mean ± standard deviation of the achieved results as a percentage. (SP-group, simulated patient group; IP-group, incognito patient group; RP-group, real patient group).

**Figure 3:**
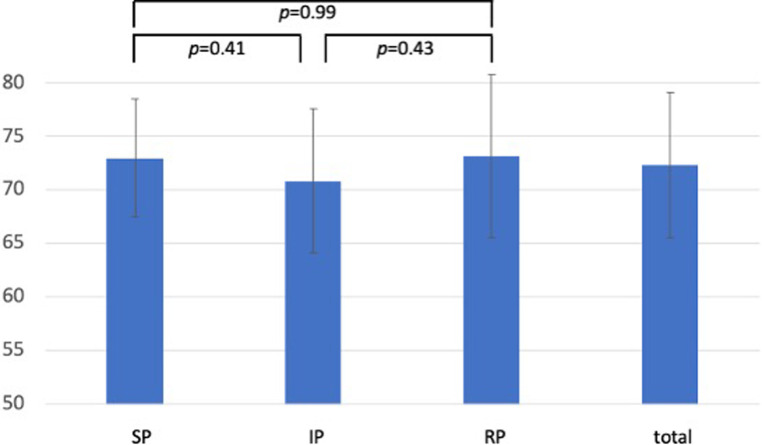
OSCE results: total rating. Results of the overall OSCE rating with the first part of the evaluation sheet weighted as two-thirds and the second part weighted as one-third of the score. Data are presented as the mean ± standard deviation of the achieved results as a percentage. (SP-group, simulated patient group; IP-group, incognito patient group; RP-group, real patient group)a.

Figures 1–3 show the results of the individual study groups in the OSCE. There were no significant differences between the three study groups in the first and second part or in the overall evaluation.

## Discussion

Since their first description in 1964 (4), SPs have been established in almost all medical schools in German-speaking countries (5). However, the influence of using SPs on the acquisition of competencies compared to RPs has not been conclusively examined.

In the present study, we showed that SPs are equivalent to RPs in terms of learning success in competency-based assessments. Thus, our results are consistent with those of McGraw et al. (15). However, in their study, 20 of 75 students were selected to train with SPs, and the remaining students formed the control group and trained with RPs. There was no difference in long-term success between the two study groups (15). However, the present study was conducted with a larger number of participants, a homogeneous group distribution, and an *a priori* sample size calculation.

Other studies reported that students described their subjective perception of the contact with RPs to be more instructive than their contact with SPs (7, 13). For example, Bokken et al. conducted a qualitative study in which medical students reported that they were better prepared for discussions with RPs than with SPs and that they found the discussions with RPs more instructive (7). On the basis of these results, we included a third study group in the present study that used incognito SPs to analyze students' subjective preference for RPs and its influence on the students' learning behavior.

We showed that there were no differences in the assessment of the content-related items or in the aspects of interaction with the patient compared to the other two study groups. Thus, the present study showed that using SPs leads to comparable results regarding the acquired competencies in taking a patient history and obtaining informed consent.

Additionally, in the present study, the IPs were not recognized by the tutors or the study participants. This finding is consistent with other studies, in which IPs are often not even recognized by the attending physicians (16–18).

Based on the principle of constructive alignment (19), the assessment of the acquired practical competencies was conducted using an assessment tool that measures the acquisition of practical competencies. However, previous studies that compared the impact of RPs and SPs used different assessment tools for their analysis. For example, Zhang et al. examined the influence of SPs on the acquisition of skills in conversational situations in gynecology (20). The students included in their study trained either with SPs, with RPs, or first with SPs and then with RPs. Their learning success was evaluated based on the analysis of the written case reports that were prepared by the students. The best results were achieved by those students who spoke to SPs, but those students who first spoke to SPs and then to RPs outperformed those who only spoke to RPs (20). Their study also indicates that conversations with SPs had a positive influence on learning success. However, the learning success was not assessed in a communication-based test, but, rather, by creating written case reports, which must be considered as a limitation of the validity of these results. Conversely, in the present study, it was possible to teach and test on the same level of competence.

Lane et al. also examined the use of SPs in teaching motivational interviewing to health care professionals, and they showed that it made no difference in terms of competence acquisition whether training was performed using an SP or *via* role play (21). In contrast to the present study, however, the participants in their study were health care professionals and not novice medical students. Medical students found the practice of speaking with SPs more useful the earlier they were in their training, which helped them to prepare for contact with RPs (7). Overall the present research suggests that it seems reasonable to integrate SPs or IPs into medical studies at an early stage so that students can derive the most benefit from their use and can initially train their communication skills in a protected environment. This appears to be particularly important in areas where sensitive issues need to be addressed, such as in urology or proctology. Practicing with SPs allows the students to first practice dealing with these topics with SPs and receive feedback regarding their behavior and handling before speaking with RPs. This method prevents careless handling of these topics that can unsettle patients or have a negative effect on the doctor–patient relationship; it also prevents the students from being disadvantaged in terms of learning success.

The positive influence of feedback from SPs on learning success has been shown in numerous studies (7, 11, 22, 23). In qualitative studies, for example, students reported that they found the feedback from SPs more useful than that from RPs (7). In addition, they reported that they receive authentic feedback-in-action through conversations with SPs, i.e. they noticed through the SP’s response during the conversation whether the conversation was going well, and thus, they were learning during the conversation (23). To prevent this from influencing the learning success in the present study, the authors decided to also recruit the RPs from the pool of SPs at Goethe University. While in their role as SPs, they received standardized role scripts and were trained for the assignment, and as RPs, they reported on their own illness and their own medical history without having received previous training or further instructions.

The present study has some limitations that need to be discussed. On the one hand, it was a single-center study within the framework of a single course curriculum. However, almost an entire semester could be included without the risk of selection bias due to participation in a voluntary, extracurricular course. The results of this study, therefore, appear to be representative and transferable to other faculties. Another limitation that needs to be discussed results from the fact that the interviews with the SPs and RPs were conducted in the skills lab. The skills lab itself is a simulated environment which holds it's own level of psychological safety. This may influence transferability to real-life scenarios. Another limitation is the choice of the curricular summative OSCE as the end point of the study because the influence of summative examinations on the learning behavior of students has been well documented (24, 25). Furthermore, there was a significant time interval between the exposure of students to SPs, IPs, or RPs during their skills lab training and the OSCE. During that time, students probably had opportunity to practice their communication skills on real patients regardless of their original exposure to SPs, IPs or RPs during the study. This may have influenced the results of the present study. Because the study was conducted as part of a well-established course curriculum, no other end point was possible. Future studies should investigate whether the results can also be verified using formative tests.

## Conclusion

Training with SPs appears to be equivalent to training with RPs in terms of competency development in communication-based assessments for surgery. Therefore, SPs should be used in the curriculum, especially at an early stage of the course. This will enable the students to practice adequate communication with patients in a protected space, especially for sensitive issues, before expanding this knowledge with RPs later in the course. Using both SPs and RPs will prevent student from experiencing disadvantages toward learning success, and it will also prevent patients from being unsettled by insensitive handling, whether conscious or unconscious, of sensitive topics.

## Data Availability

The raw data supporting the conclusions of this article will be made available by the authors, without undue reservation.
